# Meeting the International Health Regulations (2005) surveillance core capacity requirements at the subnational level in Europe: the added value of syndromic surveillance

**DOI:** 10.1186/s12889-015-1421-2

**Published:** 2015-02-07

**Authors:** Alexandra Ziemann, Nicole Rosenkötter, Luis Garcia-Castrillo Riesgo, Matthias Fischer, Alexander Krämer, Freddy K Lippert, Gernot Vergeiner, Helmut Brand, Thomas Krafft

**Affiliations:** Department of International Health, School of Public Health and Primary Care (CAPHRI), Faculty of Health, Medicine and Life Sciences, Maastricht University, P.O. Box 616, 6200 MD Maastricht, The Netherlands; Department of Medical Sciences and Surgery, Faculty of Medicine, University of Cantabria, Avenida de los Castros s/n, 39005 Santander, Spain; Department of Anaesthesia and Intensive Care, Klinik am Eichert, Postfach 660, 73006 Göppingen, Germany; Department of Public Health Medicine, School of Public Health, University of Bielefeld, P.O. Box 100131, 33501 Bielefeld, Germany; Emergency Medical Services, Head Office, Capital Region of Denmark, Telegrafvej 5, 2750 Ballerup, Denmark; Department of Clinical Medicine, Faculty of Health and Medical Sciences, University of Copenhagen, Blegdamsvej 9, 2100 Copenhagen, Denmark; Dispatch Centre Tyrol (Leitstelle Tirol Gesellschaft mbH), Hunoldstrasse 17a, 6020 Innsbruck, Austria; Institute of Environment Education and Research, Bharati Vidyapeeth University, Katraj, Dhankawadi, Satara Road, Pune, 411043 India; Institute for Geographic Sciences and Natural Resources Research, Chinese Academy of Sciences, A11 Datun Road, Beijing, 100101 China

**Keywords:** Public health surveillance, Europe, World Health Organization

## Abstract

**Background:**

The revised World Health Organization’s International Health Regulations (2005) request a timely and all-hazard approach towards surveillance, especially at the subnational level. We discuss three questions of syndromic surveillance application in the European context for assessing public health emergencies of international concern: (i) can syndromic surveillance support countries, especially the subnational level, to meet the International Health Regulations (2005) core surveillance capacity requirements, (ii) are European syndromic surveillance systems comparable to enable cross-border surveillance, and (iii) at which administrative level should syndromic surveillance best be applied?

**Discussion:**

Despite the ongoing criticism on the usefulness of syndromic surveillance which is related to its clinically nonspecific output, we demonstrate that it was a suitable supplement for timely assessment of the impact of three different public health emergencies affecting Europe. Subnational syndromic surveillance analysis in some cases proved to be of advantage for detecting an event earlier compared to national level analysis. However, in many cases, syndromic surveillance did not detect local events with only a small number of cases.

The European Commission envisions comparability of surveillance output to enable cross-border surveillance. Evaluated against European infectious disease case definitions, syndromic surveillance can contribute to identify cases that might fulfil the clinical case definition but the approach is too unspecific to comply to complete clinical definitions. Syndromic surveillance results still seem feasible for comparable cross-border surveillance as similarly defined syndromes are analysed.

We suggest a new model of implementing syndromic surveillance at the subnational level. In this model, syndromic surveillance systems are fine-tuned to their local context and integrated into the existing subnational surveillance and reporting structure. By enhancing population coverage, events covering several jurisdictions can be identified at higher levels. However, the setup of decentralised and locally adjusted syndromic surveillance systems is more complex compared to the setup of one national or local system.

**Summary:**

We conclude that syndromic surveillance if implemented with large population coverage at the subnational level can help detect and assess the local and regional effect of different types of public health emergencies in a timely manner as required by the International Health Regulations (2005).

## Background

### Diverse health threats and implementation of the International Health Regulations (2005) in Europe

Europe faces different health threats which are arising from infectious disease outbreaks, natural disasters or man-made events. The variety of potential health threats led the World Health Organization (WHO) to adjust their International Health Regulations (IHR (2005)). The IHR (2005) now follow an all-hazard approach, focusing on an “illness or medical condition, irrespective of origin or source, that presents or could present significant harm to humans” [1]. Detection, assessment and immediate reporting play an important role in the IHR (2005) and Article 5 requests every member state, within five years after the regulations came into force, to have established appropriate surveillance and response capacities [1]. Until now, surveillance in Europe primarily follows the approach of specific notifiable communicable disease reporting, which does not foresee detection or assessment of other health threats which are not defined as notifiable diseases [2]. Generally, notifiable communicable disease reporting provides data on a weekly basis. Timelier information and information on different kinds of health threats are difficult to retrieve because separate public health surveillance systems cannot be established for every kind of threat. As a result, about one third of WHO Europe member states asked for an extension of the compliance date for implementing the core capacities for public health surveillance and response with the new all-hazard requirement being seen as an obstacle [3].

### Public health surveillance in Europe – a matter of comparability

For the European Union (EU), comparability of surveillance results in the form of comparable case definitions, data formats and diagnostic codes is of high value to enable cross-border monitoring of events. EU member states, however, are very diverse and adopting comparative case definitions based on similar data sources is difficult to achieve [4]. The new directive on serious cross-border health threats 1082/2013/EU acknowledges the IHR (2005) requirements and strengthens the Union’s mandate to coordinate in times of public health crises which are potentially concerning more than one member state [5]. For communicable disease surveillance, the directive requires member states to provide information to the EU on the progression of an outbreak and about any unusual phenomena and outbreaks of unknown origin. The EU provides case definitions for comparable reporting of cases in implementing decision 2012/506/EU which member states have to use [6]. These are based on a classification of cases based on clinical signs and symptoms, laboratory, and epidemiological characteristics enabling the identification of possible, probable and confirmed cases. For threats of biological origin consisting of bio toxins or other harmful biological agents not related to communicable diseases and threats of chemical, environmental or unknown origin, member states shall inform each other based on the information from their own surveillance systems. However, the EU may adopt case definitions to which member states shall adhere [5]. The question is how member states’ surveillance capacity in terms of the IHR (2005) can be strengthened against the backdrop of both, European comparability requirements and European diversity.

### Role of the subnational level for surveillance in Europe

We understand the subnational level in this paper as primary level, e.g., county, and intermediate level, e.g., province, of the public health response in a country according to the IHR (2005) [1]. The subnational level is often the first to identify a health threat and has the responsibility to inform higher levels about an event. Also the response to an event is starting at the subnational level [7]. The IHR (2005) explicitly request the responsibility of the primary and intermediate public health response level to detect events in the whole state territory and for immediately assessing and reporting information on such events to higher levels [1].

But how can the subnational level be equipped with the means to detect different types of events or related health effects in a timely fashion? Local intelligence in the form of professionals reporting on events, e.g., in schools, or health care institutions, are the cornerstone of detecting the onset of events at the local level, as shown for example, during the 2009 A/H1N1 pandemic [8,9]. Still, there is a risk to overlook patterns that might be detected only when taking a wider perspective beyond the patients treated by one clinician or in one institution [10,11]. Surveillance systems that pool information from multiple institutions or jurisdictions can potentially detect events that are not represented in the data of any single region or institution. Using routine data, for instance from health care services, for timely detection and assessment of public health threats is in principle the idea behind the approach of syndromic surveillance.

### Syndromic surveillance – a means to meet the IHR (2005) surveillance requirements?

According to a recent definition by the European project Triple S-AGE, syndromic surveillance augments traditional surveillance systems by providing clinically nonspecific but (near) real-time information on the public health impact of events, gained from existing and if possible automatically generated data that were originally not collected for surveillance purposes [12]. The Triple S-AGE project inventoried syndromic surveillance systems in Europe and provided a detailed overview on their characteristics [13]. The project identified 124 syndromic surveillance systems worldwide and 60 in Europe [13,14]. Syndromic data sources are ranging from nonclinical sources such as web search logs, medications sales registries, and telephone helpline call logs to clinical sources such as chief complaints from primary care or emergency departments, and veterinary records [14].

Timeliness, flexibility and cost-effectiveness are considered to be the major strengths of syndromic surveillance which could make it a suitable solution for gaining timely information on different kinds of health threats as required by the IHR (2005) [15]. Its major weakness is the lack of specificity which can lead to false alerts and undetected events [16]. Therefore, the usefulness of syndromic surveillance is not undisputed, although the approach has been applied for over a decade, using different kinds of data sources and targeting various health threats [16-18]. The WHO evaluated syndromic surveillance as not applicable to become part of a global regulation to support countries to meet the IHR (2005) requirements but it was rated as potentially useful to support surveillance within countries [19]. This assessment was done back in 2001. We think it is time for a reassessment based on the evidence collated since then, and to consider a reinforced endorsement of syndromic surveillance for the support of countries’ surveillance capacity as required by the IHR (2005).

Below, we discuss the following questions around the usefulness of syndromic surveillance in the light of the IHR (2005) implementation in Europe:

1. How can syndromic surveillance be used to support European countries to meet the IHR (2005) requirements for the core capacity of immediate detection, assessment and reporting of different kinds of (potential) Public Health Emergencies of International Concern (PHEIC), especially at the subnational level?
2. How comparable are syndromic surveillance systems to enable cross-border surveillance in Europe referring to case definitions, data formats and diagnostic coding systems?
3. What are strengths and weaknesses of different implementation models for subnational syndromic surveillance in Europe?

## Discussion

### Contribution of syndromic surveillance to support European countries to meet the IHR (2005) requirements

In the following, we explore three European public health emergencies during which syndromic surveillance was applied, the 2009 A/H1N1 influenza pandemic, the volcanic ash plume which covered Europe in 2010, and the O104:H4 gastrointestinal outbreak in 2011. The pandemic was declared a PHEIC in the framework of the IHR (2005) while the other two events were reported to WHO by member states as potential PHEIC. We analyse the contribution of syndromic surveillance in the three cases in terms of timeliness, added value of subnational level application and flexibility in terms of an all-hazard approach.

#### Purpose of syndromic surveillance during three public health emergencies

During the A/H1N1 pandemic, the purpose of the application of syndromic surveillance was to detect the onset of the pandemic in a country and to gain timely information on the spatial and temporal development. Next to the syndromic surveillance systems listed in Table 1 and Table 2^a^, we identified seven additional systems^b^, for which no details were retrievable and they were not included in further analysis [13,20].

**Table 1:** Key aspects of syndromic surveillance systems assessing the A/H1N1 pandemic in Europe*

**Country**	**System name/Short description**	**Data source**	**Coding system**	**Type of syndromic ILI definition (individual, national, European, CDC)**	**Host (administrative level)**	**Potential administrative level of analysis**	**Prospective (P) or retrospective (R) data analysis**	**Reference**
Austria	SIDARTHa Tirol	Emergency medical dispatch centre	Advanced Medical Priority Dispatch System (AMPDS)	Individual	Regional	Local^†^, regional^‡^	R	[21]
Belgium	SIDARTHa Leuven	Emergency Department	Free-text	Individual	n.a.	Local	R	[21]
	SIDARTHa Belgium	Ambulance service	ICD-9	Individual	National	National	R	[21]
	Belgian absenteeism surveillance	Absenteeism	Number of absent students and workers	n.a.; (partly absenteeism due to illness)	National	Regional, national	P	[22]
Denmark	DMOS unscheduled general practitioner influenza surveillance	Primary care	ILI checkbox in electronic patient record	Close to national and European	National	Regional, national	P	[23]
France	Lyon emergency department surveillance	Emergency department	UrgIndex coding system based on free-text, ICD-10	Individual	Local	Local	R	[24]
Ireland	Out-of-hours general practitioner telephone service surveillance	Telephone helpline	Free-text	National, CDC, individual	Local	Local, regional, national	P	[25]
Italy	Liguria emergency department surveillance	Emergency department	Free-text	Close to CDC	n.a.	Local, regional	P	[26,27]
Spain	SIDARTHa Cantabria	Emergency department	Canadian Emergency Department Triage and Acuity Scale (regional adaptation)	National, close to European	n.a.	Local	R	[21,28]
Sweden	GETWELL	Web queries	Free-text	Individual, oriented at European	National	National (regional focus: Capital region)	P	[29-31]
United Kingdom	NHS Direct surveillance England/Wales	Telephone helpline	NHS Direct clinical assessment system protocol	Individual	National (England)	Local, regional, national (England, Wales)	P	[32,33]
	Q-Surveillance (scheduled care surveillance England)	Primary care	READ code	Individual	National (England)	Local, regional, national (England)	P	[32,34]
	School surveillance West Midlands	Absenteeism	n.a., number of absent students	n.a. (absenteeism due to illness)	Local	Local	R	[35]
	UK retail sales surveillance	Over-the-counter sales	No information	Individual	n.a.	Local, regional, national (England)	R	[36]
	NHS 24 surveillance Scotland	Telephone helpline	Call protocol codes, free-text	Individual	National (Scotland)	Local, regional, national (Scotland)	P	[37]
Europe	Google Flu Trends surveillance Europe	Web queries	Free-text	Individual	n.a.	National	R	[38]

*Europe = EU Member States, European Free Trade Zone countries, Acceding and Candidate countries.

^†^local = primary level of public health response in a country.

^‡^regional = intermediate level of public health response in a country.

**Table 2:** Timeliness of syndromic surveillance systems assessing the A/H1N1 pandemic in Europe*

**Country**	**System name/Short description**	**Data source**	**Reference data source**	**Timeliness [weeks]**					**Reference**
				**Aberration detection algorithm signal**	**Peak comparison (remark)**	**Cross-correlation**	**Increase comparison**	**Best result**	
Austria	SIDARTHa Tirol	Emergency medical dispatch centre	Sick leaves due to acute respiratory infections of a major Tyrolean health insurance	0				0	[21]
Belgium	SIDARTHa Leuven	Emergency Department	Sentinel general practitioners	+1.43	−1			−1	[21]
	SIDARTHa Belgium	Ambulance service	Sentinel general practitioners	0	−1	−1		−1	[21]
	Belgian absenteeism surveillance	Absenteeism	Sentinel general practitioners		−2 (work absenteeism)		−2.5 (work absenteeism)	−2.5	[24]
					−1.75 (school absenteeism)				
Denmark	DMOS unscheduled general practitioner influenza surveillance	Primary care	Laboratory confirmations, Sentinel general practitioners		−1 (sentinel general practitioners) 0 (laboratory confirmation)			−1	[23]
Spain	SIDARTHa Cantabria^†^	Emergency department	Sentinel general practitioners	+1.36	+1	+1		+1	[21]
Sweden	GETWELL^†^	Web queries	Sentinel general practitioners, Google Flu Trends		−0.5 (sentinel general practitioners) 0 (Google Flu Trends)^‡^			−0.5	[30]
United Kingdom	NHS Direct surveillance England/Wales	Telephone helpline	Q-Surveillance syndromic surveillance system				−1^‡^	n.a.	[32]
	Q-Surveillance (scheduled care surveillance England)	Primary care	NHS Direct syndromic surveillance system				+1^‡^	n.a.	[32]
	School surveillance West Midlands	Absenteeism	Laboratory confirmations, NHS Direct syndromic surveillance system		0 (sentinel general practitioners, laboratory confirmations, telephone helpline calls for cold/flu^‡^) +1 (telephone helpline calls for fever)^‡^			0	[35]
Europe	Google Flu Trends surveillance Europe	Web queries	Sentinel general practitioners		0 (7 countries)			−2	[38]
					- 1 (3 countries)				
					- 2 (1 country)				
					+1 (1 country)				
					−11 (1 country)^§^				
Average all syndromic surveillance systems								−0.56	

*Europe = EU Member States, European Free Trade Zone countries, Acceding and Candidate countries.

^†^Timeliness values for this syndromic surveillance system were calculated as average for results from two waves during the 2009 pandemic.

^‡^This value was based on reference data that cannot be defined as traditional influenza surveillance source (= other syndromic surveillance source) and was excluded from further analysis.

^§^This value was treated as outlier and was excluded from further analysis.

During the time in which the volcanic ash plume covered Europe in April 2010, syndromic surveillance was used to timely assess if there was a public health impact of the plume or rather to provide reassurance that the plume had no health effect. Next to the syndromic surveillance systems listed in Table 3, further syndromic surveillance systems were reported in Iceland and France for which we could not find any further details [39]. None of the systems produced a syndromic signal which could be connected to the ash plume. This was in line with the conclusions by WHO and the European Centre for Disease Prevention and Control (ECDC) [39,40]. During this public health emergency, syndromic surveillance systems were the only source of information to measure any direct health impact.

**Table 3:** Key aspects of syndromic surveillance systems assessing the volcanic ash plume in Europe*

**Country**	**System name/Short description**	**Data source**	**Syndromes**	**Timeliness [days]** ^**†**^	**Subnational level application (Yes = Y; No = N)**	**Prospective (P) or retrospective (R) data analysis**	**Reference**
Austria	SIDARTHa Tirol	Emergency medical dispatch	Respiratory syndrome, cardiovascular syndrome, traffic-related injuries	14	Y	R	[41]
Germany	SIDARTHa Göppingen	Ambulance service	Respiratory syndrome, cardiovascular syndrome	14	Y	R	[41]
Spain	SIDARTHa Cantabria	Emergency department	No syndrome specific analysis (only total number of cases)	14	Y	R	[41]
Sweden	GETWELL	Web queries	Not known	Not known	N	Not known	[29,31]
United Kingdom (England)	Q-Surveillance (scheduled care surveillance England)	Primary care	Asthma, conjunctivitis, allergic rhinitis, wheeze, lower respiratory tract infection, upper respiratory tract infection	1	N	P	[42]
United Kingdom (Scotland)	NHS Direct surveillance England/Wales	Telephone helpline	Difficulty breathing, eye problems, cough, rash	2	Y	P	[42]

*Europe = EU Member States, European Free Trade Zone countries, Acceding and Candidate countries.

^†^First report after first day of volcanic eruption.

The O104:H4 outbreak in 2011 originated in Northern Germany and later affected 13 countries in Europe, Canada and the United States of America. The outbreak first developed unnoticed for around two weeks mainly because of time lost in the information flow from local to regional and national level in the decentralised epidemiological reporting system in Germany [43]. Upon becoming aware of the outbreak, the German national centre for disease control enhanced the frequency of reporting from subnational to national level to daily reporting and implemented a syndromic surveillance system for bloody diarrhoea in emergency departments [44]. We did not identify reports of syndromic surveillance use in any of the other affected countries during this public health emergency.

#### Timeliness of syndromic surveillance during three public health emergencies

During the influenza pandemic, the syndromic surveillance systems provided information on the onset or peak of the pandemic on average half a week before traditional surveillance systems, at the earliest (Table 2). These timeliness assessments did not take into account the common reporting delay of traditional sentinel influenza surveillance systems. They refer to an earlier detection of cases analysing data sources in which (potential) influenza patients are registered earlier in the course of illness or treatment, as compared to traditional data collected later in the process by sentinel general practitioners or laboratories. Additional time is gained because syndromic surveillance information usually is available daily while sentinel and laboratory information often is reported up to a week after the case was registered [21,23,35]. Many syndromic systems were already established and were ready to be used when the pandemic occurred which saved time in comparison to ad-hoc set up systems.

During the volcanic ash plume event, information was made available to the English public one day after the first day of eruption and two days afterwards in Scotland. Information was updated in the following days and weeks [42]. The first information from the systems in Austria, Germany, and Spain was available to the public two weeks after the first day of eruption and was updated again 10 days later [41] (Table 1). There is no information on the timeliness for the syndromic surveillance system in Sweden. As there were no other health surveillance systems used during the ash plume, there is no gold standard to compare the timeliness of syndromic surveillance systems to.

During the O104:H4 outbreak, the syndromic surveillance system was implemented on 27 May, 8 days after Robert Koch-Institute was notified of a first cluster of cases and 5 days after the EU and WHO were informed. The system was terminated on 30 September 2011. Syndromic reporting was daily. The syndromic surveillance system was set up ad-hoc, relied on manual data collection and reporting (via fax or email) and was voluntary. Thus, syndromic reporting varied in completeness and continuity [43].

In synthesis, syndromic surveillance provided timely information during the three events supporting the IHR (2005) requirement of immediate assessment and reporting. Also during other subnational and national emergencies, syndromic surveillance provided timelier information, as for example found in a review by Dailey et al. [45]. For rare or non-communicable health threats such as the volcanic ash plume, there are often no established health surveillance systems. As clinically specific systems cannot be implemented for every conceivable health threat like the volcanic ash plume, syndromic surveillance is often the only source of timely information [46].

#### Added value of syndromic surveillance at the subnational level during three public health emergencies

During the influenza pandemic, 13 out of 15 systems provided syndromic information at the subnational level, while nine systems and the European study provided data at the national level (Table 1). Smith et al. [32], Kavanagh et al. [37], and Todd et al. [36] highlighted the use of local analyses for earlier identification of the onset of the pandemic in certain regions compared to the national level analysis. Rosenkötter et al. compared among others ambulance patient records at the local level in one country with national level data in another and found better validity for national level data [21]. The low validity at the local level was explained by the difficulty to differentiate signal from noise when very small case numbers were analysed. The authors also compared single emergency departments located at the subnational level in two different countries and found that both provided syndromic data of sufficient validity and timeliness compared to sentinel data in the regions. Case numbers at these emergency departments were large enough for a sound syndromic surveillance analysis. These examples show that a valid application at the subnational level is achievable if the analysed data source can potentially reflect a critical mass of cases.

During the volcanic ash plume event, information in Sweden [29] and the United Kingdom (UK) was only published for the national level, i.e., England and Scotland, however, in Scotland also the subnational level was analysed but no results were presented [42]. The systems in Austria, Germany and Spain were providing information at the subnational level [41]. As during the influenza pandemic, the systems in Austria and Germany were analysing low case numbers which was affecting the validity of the results.

During the O104:H4 outbreak, 193 German emergency departments participated in total, of which 28 were located in the more effected areas. The subnational syndromic data were aggregated to county level and were assessed as suitable for the timely analysis of the development of the outbreak [44].

In conclusion, syndromic surveillance can support public health authorities at the subnational level to detect events earlier or rapidly gain information about the health impact of an event. Although many syndromic surveillance systems are applied at the subnational level, the added value compared to a national application is often not analysed. The problem of distinguishing signals from noise, especially of events with low case numbers, also became apparent during other syndromic surveillance applications at subnational or national level [47,48].

#### All-hazard applicability of syndromic surveillance

The examples showed that syndromic surveillance was used to assess the health impact of different (potential) communicable and non-communicable PHEIC which is in line with the all-hazard requirement of the IHR (2005). Although these are only three examples, the flexibility of syndromic surveillance to analyse different kinds of health threats was shown during many subnational or national level applications in Europe, such as mass-gatherings, e.g., the Olympic Games in the UK, environmental events, e.g., heat and cold waves or floods, and diverse communicable disease outbreaks [13,20]. Also Paterson and colleagues highlighted the “remarkable adaptability of syndromic surveillance” in their recent review [15].

### Comparability of syndromic surveillance output for cross-border surveillance in Europe

We explore the European comparability of syndromic surveillance systems, especially in terms of compliance to European case definitions used during the A/H1N1 influenza pandemic and the O104:H4 outbreak [6].

During the influenza pandemic, the 15 systems and the European study analysed influenza-like illness (ILI) or respiratory symptoms based on different case definitions (Table 1). Some systems applied the Centers for Disease Control and Prevention (CDC) definition for ILI [26,49] or the European ILI definition established by ECDC [28] which are based on symptoms. Others use aggregated diagnostic information and not symptoms, self-defined queries of standardised coding categories or free-text. Also for syndromic surveillance systems based on the same group of data sources, for instance emergency departments, the diagnostic coding systems differ. It might not be achievable to use the European definition in a syndromic surveillance system because the underlying source does not provide all necessary information. For example, the use of the European definition was attempted for the syndromic surveillance system in Sweden but the analysed data source of web queries did not provide all necessary information [29]. For the system in Ireland, different definitions for free-text searches were compared, one being based on the national definition, another one on the CDC definition, but the best outcome for identifying influenza cases was achieved using a definition which was tailored to the available information in the data source [25]. Other syndromic surveillance systems do not use a definition but analyse the total volume which is reducing specificity, for example systems using school absenteeism data [35,50,51]. These examples show that not one and the same case definition can and should be applied when using syndromic surveillance in order to achieve valid results. Although this means that these case definitions are not comparable, most systems analysed similar syndromes indicating ILI.

None of the examples provided evidence that syndromic surveillance is capable of identifying an A/H1N1 case according to the full European clinical case definition. The syndromic surveillance systems could help identify cases meeting a part of the clinical case definition, always depending on the information provided by the analysed data source.

The EU did not provide any case definition during the volcanic ash plume event. The cross-border comparability of the definitions used by the different syndromic surveillance systems is to be considered weak due to differences in the analysed data sources as seen also during the A/H1N1 pandemic.

During the O104:H4 outbreak, the syndromic surveillance output could not meet the European clinical case definition of *Shiga* Toxin-Producing Escherichia coli (STEC)-associated Haemolytic Uremic Syndrome (HUS), only for STEC/Verotoxin producing Escherichia coli related diarrhoea. Therefore, syndromic surveillance could not contribute to clinical STEC-associated HUS case classification. However, the European comparability of the syndrome “bloody diarrhoea” can be considered high as it is based on a relatively simply defined symptom which is clearly distinguishable from other symptoms.

To conclude, syndromic surveillance can contribute to identifying clinical cases according to the European case definitions but it is unlikely that syndromic information provides more detailed information to identify cases according to full clinical case definitions as required by the IHR (2005). Syndromic surveillance is rather suited to augment existing surveillance systems in order to provide a timely indication of clinical cases to be verified by traditional surveillance and assessment measures.

The low level of comparability of syndromic information in Europe in terms of case definitions, data formats and diagnostic coding does not speak for implementing standardised syndromic surveillance systems at European level or for aiming at a harmonisation of existing systems in order to support cross-border surveillance. From a pragmatic point of view one could argue that the systems are analysing similarly defined syndromes, such as ILI, and are in this way enabling cross-border surveillance. Also the European influenza surveillance system is based on different data sources and different case definitions of ILI or acute respiratory illness [52-54].

Following this line of argument, the Triple S-AGE project suggested three models of syndromic surveillance harmonization in Europe: (A) a European syndromic surveillance system model with standardised data sources and case definitions as implemented for mortality monitoring in the European Mortality Monitoring system [55], (B) a completely disharmonised European model as it is existing at the moment for all morbidity syndromic surveillance systems, and (C) an intermediate model with diverse data sources and case definitions but standardised reporting at the European level [56]. Depending on the characteristics of the syndrome/event and the opportunity to generate comparable syndromes from the available data, these models can exist in parallel. From a European point of view, the goal might be to find the optimal combination of these models with the largest possible harmonisation to allow for cross-border comparison. In the following section, we will look at the strengths and weaknesses of different implementation models of syndromic surveillance at the subnational level in Europe (following the Triple S-AGE models B and C).

### Strengths and weaknesses of implementation models for subnational syndromic surveillance in Europe

#### Current subnational syndromic surveillance implementation: national vs. local model

The implementation of syndromic surveillance systems for subnational surveillance in European countries currently follows either a national or a local model (Table 1). Syndromic surveillance systems following the national model are hosted at the national level, collecting subnational data, collating and analysing it at the national level, and reporting surveillance results at the national and subnational level. Examples for national syndromic surveillance systems in Europe are especially found in the UK [32,34,37,50,57] and France [58,59], which are the longest established syndromic surveillance systems in Europe. This might be explained by the fact that both countries have centrally organised health systems [20]. Implementation of national systems is likely to be limited in federal countries with a long tradition of predominantly decentralised health systems [10].

Syndromic surveillance systems following the local model are hosted at the local level, collating and analysing data covering a single jurisdiction and reporting surveillance results for that jurisdiction only. Examples for local syndromic surveillance systems are found in many countries (Table 1), also in countries having national syndromic surveillance systems, e.g., in Italy [26,60], and the UK [35,61].

Table 4 compares the strengths and weaknesses of the two models for some syndromic surveillance system characteristics for which we could identify an impact by the model of implementation. The characteristics are defined following the framework for assessing syndromic surveillance systems proposed by the CDC [62] and detailed by the Triple S-AGE project [63].

**Table 4:** Strengths and weaknesses of syndromic surveillance system implementation models in Europe*

**Syndromic surveillance system characteristics**	**National model** ^**†**^	**Local model** ^**‡**^	**Integrated subnational model** ^**§**^
Simplicity and costs of setup	++	+	--
Simplicity of access to subnational data sources	--	+	+
Stability: Potential of single system failure in times of crisis	--	--	++
Acceptance and utilization of syndromic surveillance results at subnational level	--	++	++
Flexibility of adjustment to local events/priorities	--	++	++
Data protection problems	--	++	++
Data quality	--	++	++
Validity: Interpretation of signals including false alerts (signal-to-noise problem)	--	+	+
Validity: Small-number problem in detecting local events	--	--	--
Validity: Detection of events covering multiple local jurisdictions	+	--	+
Representativeness of whole country	+	--	++
Comparability of surveillance results across multiple subnational jurisdictions	++	--	+
Transferability between subnational jurisdictions	+	--	+
Clinical resource and quality management in health care institutions	--	+	+
Crisis preparedness of health care institutions	--	+	+

*Europe = EU Member States, European Free Trade Zone countries, Acceding and Candidate countries.

^†^Data collation and analysis at national level, representing several subnational jurisdictions, top-down reporting to national, regional and local level.

^‡^Data collation and analysis at local level, representing a single subnational jurisdiction, local reporting to local level.

^§^Data collation and analysis at local level, analysis of aggregated data at regional or national level representing several subnational jurisdictions, standardised bottom-up reporting to local, regional and national level.

National systems have the biggest advantage in simplicity and cost-effectiveness. To set up a syndromic surveillance system at the national level without involving too many stakeholders, based on national registries or a national point of access to a network of data providers is relatively easy. Examples for such systems are found in Belgium [21], France [64] or the UK [36]. If data collection is organised regionally or different data collection software is used as it is often the case for health care services, access to data sources can be difficult, impeding representativeness of the whole country and delaying setup of a system, as reported for example for syndromic surveillance systems in England [20].

Comparability of surveillance system results in national systems is high as data source, case definitions, analysis methodology, and reporting are the same for the whole country. The chance of detecting events covering multiple subnational jurisdictions is high in a national system and impossible in a local system. The signal-to-noise problem of syndromic surveillance which limits detection of events with small case numbers is the same for both models.

A single national or local syndromic surveillance system may be prone to become a “single point of failure” in times of crises, because of a power failure, for example [7]. A decentralised surveillance system can provide information during an event even if parts of the system are not working.

The biggest advantage of the local syndromic surveillance model is that it can be adjusted to the local circumstances of data availability and accessibility, data collection procedures, treatment seeking behaviour of the population and priorities for targeted health threats. Syndromic surveillance performs best if fine-tuned to the characteristics of the analysed data source as shown for the influenza syndromic surveillance system in Spain, for example [28].

A local system which is operated by the professionals responsible for local surveillance is more easily “owned” by these professionals and will foster motivation and quality of work, compared to a system which is imposed by the national level [20,65].

A close collaboration between local public health authorities and local health care institutions which often function as data providers can simplify the setup and maintenance of syndromic surveillance systems. Data providers know their data and the context of data collection best. They can identify the most suitable data fields for analysis which improves data quality, and they can support interpretation of syndromic surveillance results by giving explanations for signals. Collaboration between health care institutions and public health authorities for a syndromic surveillance system can enhance collaboration of these two often divided parts of the health system for crisis preparedness [10]. Finally, syndromic surveillance output based on health care data can also be useful to support clinical resource management in health care institutions during times of crisis [8].

Another advantage of local systems is that raw data are analysed in the same local jurisdiction compared to a national system where data are leaving the jurisdiction to be analysed at the national level. This can reduce problems of data privacy that are arising from analysing patient information [66].

In summary, there are distinct advantages and disadvantages of the national and the local syndromic surveillance system models which are hampering to make the best use of syndromic surveillance. Systems should at best be set up locally adjusted but also covering a large part of the population. In the following we propose a system model that combines these advantages of the national and local system model.

#### A new model: Subnational syndromic surveillance implementation

We suggest a new implementation model in which syndromic surveillance is integrated in existing surveillance structures at the subnational level. Data collation and analysis are done at the local level, while higher levels can analyse aggregated syndromic data or only receive reports. This is to be decided in each country and will depend on the organisation of the public health and surveillance system. Transferring aggregated instead of raw data or reports to higher levels has the advantage that data privacy rules are respected. Reporting of surveillance results for the local, regional and national level is following a standardised format. The approach can only live up to its potential by covering a large part of the population and several jurisdictions.

**Figure 1:**
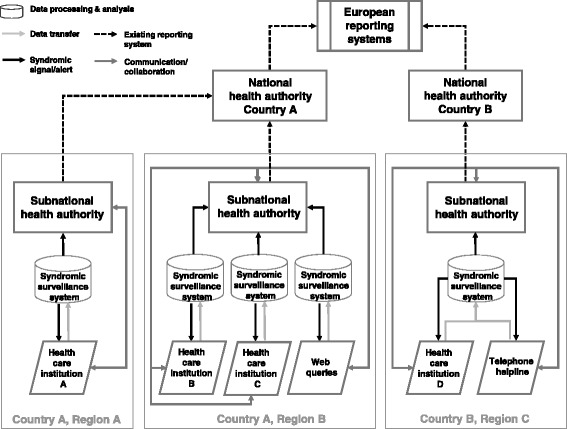
**The SIDARTHa model for integrated syndromic surveillance at the subnational level.** SIDARTHa syndromic surveillance systems are implemented at subnational level and can be based on one or different kinds of data sources. In this way, the data analysis algorithms can be chosen and adjusted according to the immediate context. The syndromic surveillance results feed into the established surveillance and reporting system of the responsible subnational health authority augmenting existing (traditional) surveillance information. Syndromic information would only be reported to higher levels in aggregated form limiting problems arising from data privacy. Investigation of signals is done at subnational level but could also be done at national levels to allow for detection of events covering several jurisdictions. The data providing institutions should also receive access to syndromic surveillance results for their institution and/or jurisdiction which could be used by them for resource planning purposes.

In the EU project “European Emergency Data Based System for Information on, Detection and Analysis of Risks and Threats to Health” (SIDARTHa), we developed a subnational syndromic surveillance implementation model based on three different emergency care data sources [67]. Figure 1 depicts the setup, data flow and reporting directions in a SIDARTHa syndromic surveillance system. A decision tree was developed for the SIDARTHa approach for validation of signals so that not every syndromic signal results in a public health response [68]. Currently, the SIDARTHa system is implemented in one region in the countries of Austria (active), Belgium (pilot), Germany (pilot) and Spain (active) [21,68].

Table 4 compares the strengths and weaknesses of the subnational model compared to the local and national model. The model combines the advantages of the local and the national model for most characteristics. An additional improvement of this model is the stability in times of crisis by the decentralised setup of systems. Further, representativeness is anticipated to be increased through easier access to subnational data sources by subnational surveillance system operators. However, comparability of surveillance results in terms of case definitions, data formats and diagnostic coding systems can be anticipated lower as in a national system setup. Systems following the subnational model are likely to differ in their setup because they are adjusted to the local accessibility and characteristics of data sources in each region. The comparability can be increased if a common framework is used for setting up a system, as for example developed by the European projects SIDARTHa and Triple S-AGE.

The major disadvantage in comparison to the local and national model is the higher effort and complexity in the setup of a decentralised and locally adjusted system. Because of the high effort, the two active SIDARTHa systems in Austria and Spain could only be implemented as local systems providing information for one region in the respective country. For a roll-out to other regions we consider political endorsement and support from higher levels as vital.

## Summary

We conclude that syndromic surveillance can support countries to detect and assess the public health impact of different types of PHEIC at the subnational level as stipulated in the IHR (2005) core surveillance capacity requirements. The approach provided timely information during three different public health emergencies in Europe. For some events syndromic surveillance systems were the only available source of near real-time information. Many syndromic surveillance systems are applied at the subnational level, which in some cases proved to be of advantage for detecting an event earlier compared to the national level. Syndromic surveillance is not suited to detect local events consisting of small case numbers.

In terms of case definitions, data formats or diagnostic coding systems, syndromic surveillance systems are not identical across Europe because of the diversity of the analysed data sources. Nevertheless, we consider comparable cross-border surveillance possible based on similarly defined syndromes.

Implementation of syndromic surveillance in Europe currently follows either a local or national model. In order to gain the most of syndromic surveillance we suggest a new subnational approach of implementing syndromic surveillance. The model foresees locally adjusted data collation and analysis at the subnational level, and integrated and standardised reporting to higher levels. By covering a large part of the population, events covering several jurisdictions can be identified at higher levels. However, the setup of decentralised and locally adjusted systems is more complex compared to setup of one national or local system.

Using guidelines and tools produced by European syndromic surveillance projects and with national or European policy support, a wider roll-out of syndromic surveillance across Europe can be achieved. Only by expanding the application of syndromic surveillance, European countries will be positioned to timely assess the public health impact of potential PHEIC, especially rare and non-communicable events.

## Endnotes

^a^The literature search was accomplished in June 2013 and updated in July 2014. The search string for PubMed was: (H1N1[Title/Abstract] OR pandemic[Title/Abstract]) AND “syndromic surveillance”[Title/Abstract]. The search string for Google Scholar was: H1N1 OR pandemic AND “syndromic surveillance”. The review of Google Scholar hits stopped after 10 pages of hits which did not provide any new relevant content. We checked references of selected full-text articles for further relevant publications.

^b^The additional identified systems were the SurSauUD emergency department and general practitioner house calls surveillance systems in France, the national emergency department surveillance system and the Lazio emergency department surveillance system in Italy, the South Holland South general practitioner pandemic surveillance system in the Netherlands, the PIPeR general practitioner pandemic surveillance system in Scotland, the general practitioner out-of-hours surveillance system in Ireland, and the general practitioner surveillance system in Wales.

## Abbreviations

AMPDS: Advanced Medical Priority Dispatch System
CDC: Centers for Disease Control and Prevention
ECDC: European Centre for Disease Prevention and Control
EU: European Union
ILI: Influenza-like illness
IHR(2005): International health regulations 2005 revision
HUS: Haemolytic uremic syndrome
PHEIC: Public Health Emergency of International Concern
SIDARTHa: European Emergency Data Based System for Information on, Detection and Analysis of Risks and Threats to Health
STEC: *Shiga* toxin-producing Escherichia coli
Triple S-AGE: Syndromic Surveillance Survey, Assessment towards Guidelines for Europe
UK: United Kingdom
WHO: World Health Organization

## References

[1] World Health Organization (2008). International Health Regulations (2005).

[2] Paquet C, Coulombier D, Kaiser R, Ciotti M (2006). Epidemic intelligence: a new framework for strengthening disease surveillance in Europe. Euro Surveill.

[3] World Health Organization (2013). European Strategy Meeting for implementation of the International Health Regulations (2005) - Scope and purpose.

[4] Allebeck P (2012). Which health data for Europe?. Eur J Public Health.

[5] European Parliament and Council of the European Union (2013). Decision on serious cross-border threats to health and repealing Decision No 2119/98/EC (1082/2013/EU). Off J Eur Union.

[6] European Commission (2012). Commission Implementing Decision amending Decision 2002/253/EC laying down case definitions for reporting communicable diseases to the Community network under Decision No 2119/98/EC of the European Parliament and of the Council (2012/506/EU). Off J Eur Union.

[7] Mostashari F, Hartman J (2003). Syndromic surveillance: a local perspective. J Urban Health.

[8] McManus J, Huebner K, Scheulen J (2006). The science of surge: Detection and situational awareness. Acad Emerg Med.

[9] Lyytikainen O, Kuusi M, Snellman M, Virtanen M, Eskola J, Ronkko E (2011). Surveillance of influenza in Finland during the 2009 pandemic, 10 May 2009 to 8 March 2010. Euro Surveill.

[10] Wilson K, McDougall C, Forster A (2009). The responsibility of healthcare institutions to protect global health security. Healthc Q.

[11] Altmann M, Spode A, Altmann D, Wadl M, Benzler J, Eckmanns T (2011). Timeliness of surveillance during outbreak of Shiga Toxin-producing Escherichia coli infection, Germany, 2011. Emerg Infect Dis.

[12] Triple S (2011). Assessment of syndromic surveillance in Europe. Lancet.

[13] Conti S, Kanieff M, Rago G. Inventory of syndromic surveillance systems in Europe. Triple S-AGE project; 2012. [http://www.syndromicsurveillance.eu/triple-s_inventory_report.pdf]

[14] Ziemann A, Krafft T. Guidelines for assessment of data sources. Triple S-AGE project; 2013. [http://www.syndromicsurveillance.eu/triple-s_guidelines_datasources.pdf]

[15] Paterson BJ, Durrheim DN (2013). The remarkable adaptability of syndromic surveillance to meet public health needs. J Epidemiol Global Health.

[16] Kaydos-Daniels SC, Rojas Smith L, Farris TR (2013). Biosurveillance in outbreak investigations. Biosecur Bioterror.

[17] Koopmans M (2013). Surveillance strategy for early detection of unusual infectious disease events. Curr Opin Virol.

[18] Morse SS (2012). Public health surveillance and infectious disease detection. Biosecur Bioterror.

[19] Revision of the International Health Regulations (2001). Progress report, February 2001. Wkly Epidemiol Rec.

[20] Ziemann A, Krafft T, Sala Soler M, Sypniewska P. Country visits. Triple S-AGE project; 2013 [http://syndromicsurveillance.eu/images/stories/Final_material/triple-S_country_visits.pdf]

[21] Rosenkötter N, Ziemann A, Garcia-Castrillo Riesgo L, Gillet JB, Vergeiner G, Krafft T (2013). Validity and timeliness of syndromic influenza surveillance during the autumn/winter wave of A(H1N1) influenza 2009: results of emergency medical dispatch, ambulance and emergency department data from three European regions. BMC Public Health.

[22] Bollaerts K, Antoine J, Robesyn E, Van Proeyen L, Vomberg J, Feys E (2010). Timeliness of syndromic influenza surveillance through work and school absenteeism. Arch Public Health.

[23] Harder KM, Andersen PH, Baehr I, Nielsen LP, Ethelberg S, Glismann S (2011). Electronic real-time surveillance for influenza-like illness: experience from the 2009 influenza A(H1N1) pandemic in Denmark. Euro Surveill.

[24] Gerbier-Colomban S, Potinet-Pagliaroli V, Metzger MH (2014). Can epidemic detection systems at the hospital level complement regional surveillance networks: case study with the influenza epidemic?. BMC Infect Dis.

[25] Brabazon ED, Carton MW, Murray C, Hederman L, Bedford D (2010). General practice out-of-hours service in Ireland provides a new source of syndromic surveillance data on influenza. Euro Surveill.

[26] Ansaldi F, Orsi A, Altomonte F, Bertone G, Parodi V, Carloni R (2008). Emergency department syndromic surveillance syndromic surveillancetem for early detection of 5 syndromes: a pilot project in a reference teaching hospital in Genoa. Italy. J Prev Med Hyg.

[27] Amicizia D, Cremonesi I, Carloni R, Schiaffino S (2011). The response of the Liguria Region (Italy) to the pandemic influenza virus A/H1N1sv. J Prev Med Hyg.

[28] Schrell S, Ziemann A, Garcia-Castrillo Riesgo L, Rosenkotter N, Llorca J, Popa D (2013). Local implementation of a syndromic influenza surveillance syndromic surveillancetem using emergency department data in Santander, Spain. J Public Health (Oxf).

[29] Hulth A, Rydevik G, Linde A (2009). Web queries as a source for syndromic surveillance. PLoS One.

[30] Hulth A, Rydevik G (2011). Web query-based surveillance in Sweden during the influenza A(H1N1)2009 pandemic, April 2009 to February 2010. Euro Surveill.

[31] Hulth A, Rydevik G (2011). GET WELL: an automated surveillance syndromic surveillancetem for gaining new epidemiological knowledge. BMC Public Health.

[32] Smith S, Smith GE, Olowokure B, Ibbotson S, Foord D, Maguire H (2011). Early spread of the 2009 influenza A(H1N1) pandemic in the United Kingdom--use of local syndromic data, May-August 2009. Euro Surveill.

[33] Cooper DL, Verlander NQ, Elliot AJ, Joseph CA, Smith GE (2009). Can syndromic thresholds provide early warning of national influenza outbreaks?. J Public Health (Oxf).

[34] Harcourt SE, Smith GE, Elliot AJ, Pebody R, Charlett A, Ibbotson S (2012). Use of a large general practice syndromic surveillance syndromic surveillancetem to monitor the progress of the influenza A(H1N1) pandemic 2009 in the UK. Epidemiol Infect.

[35] Kara EO, Elliot AJ, Bagnall H, Foord DG, Pnaiser R, Osman H (2012). Absenteeism in schools during the 2009 influenza A(H1N1) pandemic: a useful tool for early detection of influenza activity in the community?. Epidemiol Infect.

[36] Todd S, Diggle PJ, White PJ, Fearne A, Read JM (2014). The spatiotemporal association of non-prescription retail sales with cases during the 2009 influenza pandemic in Great Britain. BMJ Open.

[37] Kavanagh K, Robertson C, Murdoch H, Crooks G, McMenamin J (2012). Syndromic surveillance of influenza-like illness in Scotland during the influenza A H1N1v pandemic and beyond. J R Stat Soc (Series A).

[38] Valdivia A, Lopez-Alcalde J, Vicente M, Pichiule M, Ruiz M, Ordobas M (2010). Monitoring influenza activity in Europe with Google Flu Trends: comparison with the findings of sentinel physician networks - results for 2009–10. Euro Surveill.

[39] European Centre for Disease Prevention and Control (2010). Second Interim Threat Assessment. Ash cloud following volcanic eruption in Iceland, 20 April 11:00 CET.

[40] World Health Organization Regional Office for Europe. WHO/Europe expert group concludes Icelandic volcanic ash currently poses no threat to public health. [http://www.euro.who.int/en/health-topics/environment-and-health/air-quality/news/news/2010/05/whoeurope-expert-group-concludes-icelandic-volcanic-ash-currently-poses-no-threat-to-public-health]

[41] Rosenkötter N, Ziemann A, Garcia-Castrillo Riesgo L, Vergeiner G, Fischer M, Krafft T (2010). SIDARTHa Volcanic Ash Cloud Rapid Public Health Impact Assessment. Regional public health impact of volcanic ash cloud covering Europe after eruption of Eyjafjallajoekull, Iceland starting April 14th, 2010. Results as of May 15th, 2010.

[42] Elliot AJ, Singh N, Loveridge P, Harcourt S, Smith S, Pnaiser R (2010). Syndromic surveillance to assess the potential public health impact of the Icelandic volcanic ash plume across the United Kingdom, April 2010. Euro Surveill.

[43] Robert Koch-Institut (2011). Final presentation and evaluation of epidemiological findings in the EHEC O104:H4 Outbreak, Germany 2011.

[44] Wadl M, Rieck T, Nachtnebel M, Greutelaers B, an der Heiden M, Altmann D (2011). Enhanced surveillance during a large outbreak of bloody diarrhoea and haemolytic uraemic syndrome caused by Shiga toxin/verotoxin-producing Escherichia coli in Germany, May to June 2011. Euro Surveil.

[45] Dailey L, Watkins RE, Plant AJ (2007). Timeliness of data sources used for influenza surveillance. J Am Med Inform Assoc.

[46] Rosenkötter N, Ziemann A, Krafft T, Riesgo LG, Vergeiner G, Brand H (2014). Non-infectious events under the International Health Regulations (2005) in Europe - a case for syndromic surveillance. J Public Health Policy.

[47] Coory MD, Kelly H, Tippett V (2009). Assessment of ambulance dispatch data for surveillance of influenza-like illness in Melbourne, Australia. Public Health.

[48] Xing J, Burkom H, Tokars J (2011). Method selection and adaptation for distributed monitoring of infectious diseases for syndromic surveillance. J Biomed Inform.

[49] van den Wijngaard C, van Asten L, van Pelt W, Nagelkerke NJ, Verheij R, de Neeling AJ (2008). Validation of syndromic surveillance for respiratory pathogen activity. Emerg Infect Dis.

[50] Mook P, Joseph C, Gates P, Phin N (2007). Pilot scheme for monitoring sickness absence in schools during the 2006/07 winter in England: can these data be used as a proxy for influenza activity?. Euro Surveill.

[51] Schmidt WP, Pebody R, Mangtani P (2010). School absence data for influenza surveillance: a pilot study in the United Kingdom. Euro Surveill.

[52] Aguilera JF, Paget WJ, Mosnier A, Heijnen ML, Uphoff H, van der Velden J (2003). Heterogeneous case definitions used for the surveillance of influenza in Europe. Eur J Epidemiol.

[53] Paget J, Marquet R, Meijer A, van der Velden K (2007). Influenza activity in Europe during eight seasons (1999–2007): an evaluation of the indicators used to measure activity and an assessment of the timing, length and course of peak activity (spread) across Europe. BMC Infect Dis.

[54] Kissling E, Valenciano M, Falcao J, Larrauri A, Widgren K, Pitigoi D (2009). “I-MOVE” towards monitoring seasonal and pandemic influenza vaccine effectiveness: lessons learnt from a pilot multi-centric case–control study in Europe, 2008–9. Euro Surveill.

[55] EuroMOMO. [http://www.euromomo.eu]

[56] Medina S, Fouillet A, Ziemann A, Krafft T, Cooper D, Dupuy C, et al. Proposal for a European strategy for syndromic surveillance. Toward comparability of reporting from syndromic surveillance systems in Europe. Triple S-AGE project. 2013 [http://syndromicsurveillance.eu/Triple-S_proposal.pdf].

[57] Cooper DL, Smith G, Baker M, Chinemana F, Verlander N, Gerard E (2004). National symptom surveillance using calls to a telephone health advice service–United Kingdom, December 2001-February 2003. MMWR Morb Mortal Wkly Rep.

[58] Flamand C, Larrieu S, Couvy F, Jouves B, Josseran L, Filleul L (2008). Validation of a syndromic surveillance syndromic surveillancetem using a general practitioner house calls network, Bordeaux, France. Euro Surveill.

[59] Josseran L, Nicolau J, Caillere N, Astagneau P, Brucker G (2006). Syndromic surveillance based on emergency department activity and crude mortality: two examples. Euro Surveill.

[60] Guasticchi G, Giorgi Rossi P, Lori G, Genio S, Biagetti F, Gabriele S (2009). Syndromic surveillance: sensitivity and positive predictive value of the case definitions. Epidemiol Infect.

[61] Davies GR, Finch RG (2003). Sales of over-the-counter remedies as an early warning syndromic surveillancetem for winter bed crises. Clin Microbiol Infect.

[62] Buehler JW, Hopkins RS, Overhage JM, Sosin DM, Tong V (2004). Framework for evaluating public health surveillance systems for early detection of outbreaks: recommendations from the CDC Working Group. MMWR Recomm Rep.

[63] Ziemann A, Krafft T. Scientific visit guidelines for knowledge exchange on syndromic surveillance in Europe. Triple S-AGE project. 2011 [http://syndromicsurveillance.eu/triple-s_scientific_visit_guidelines.pdf]

[64] Bounoure F, Beaudeau P, Mouly D, Skiba M, Lahiani-Skiba M (2011). Syndromic surveillance of acute gastroenteritis based on drug consumption. Epidemiol Infect.

[65] Buehler JW, Whitney EA, Smith D, Prietula MJ, Stanton SH, Isakov AP (2009). Situational uses of syndromic surveillance. Biosecur Bioterror.

[66] EuroREACH. Good practice on data linkages and performance measurement in relation to access to national health care data systems*.* [http://www.euroreach.net/sites/default/files/EuroREACH-WP3%20Final%20Report.pdf]

[67] SIDARTHa. [http://www.sidartha.eu]

[68] Ziemann A, Rosenkötter N, Garcia-Castrillo Riesgo L, Schrell S, Kauhl B, Vergeiner G (2014). A concept for routine emergency-care data-based syndromic surveillance in Europe. Epidemiol Infect.

